# Small-molecule inhibitors of P-Rex guanine-nucleotide exchange factors

**DOI:** 10.1080/21541248.2022.2131313

**Published:** 2022-11-07

**Authors:** CD Lawson, K Hornigold, D Pan, I Niewczas, S Andrews, J Clark, HCE Welch

**Affiliations:** a Signalling Programme, The Babraham Institute, Babraham Research Campus, Cambridge CB22 3AT, UK; b Biological Chemistry Facility, The Babraham Institute, Babraham Research Campus, Cambridge CB22 3AT, UK; c Bioinformatics Facility, The Babraham Institute, Babraham Research Campus, Cambridge CB22 3AT, UK

**Keywords:** P-Rex1, P-Rex2, PREX1, PREX2, rac, guanine-nucleotide exchange factor (GEF), Rac-GEF, Rho-GEF, inhibition, small-molecule inhibitor

## Abstract

P-Rex1 and P-Rex2 are guanine-nucleotide exchange factors (GEFs) that activate Rac small GTPases in response to the stimulation of G protein-coupled receptors and phosphoinositide 3-kinase. P-Rex Rac-GEFs regulate the morphology, adhesion and migration of various cell types, as well as reactive oxygen species production and cell cycle progression. P-Rex Rac-GEFs also have pathogenic roles in the initiation, progression or metastasis of several types of cancer. With one exception, all P-Rex functions are known or assumed to be mediated through their catalytic Rac-GEF activity. Thus, inhibitors of P-Rex Rac-GEF activity would be valuable research tools. We have generated a panel of small-molecule P-Rex inhibitors that target the interface between the catalytic DH domain of P-Rex Rac-GEFs and Rac. Our best-characterized compound, P-Rex inhibitor 1 (PREX-in1), blocks the Rac-GEF activity of full-length P-Rex1 and P-Rex2, and of their isolated catalytic domains, *in vitro* at low-micromolar concentration, without affecting the activities of several other Rho-GEFs. PREX-in1 blocks the P-Rex1 dependent spreading of PDGF-stimulated endothelial cells and the production of reactive oxygen species in fMLP-stimulated mouse neutrophils. Structure-function analysis revealed critical structural elements of PREX-in1, allowing us to develop derivatives with increased efficacy, the best with an IC_50_ of 2 µM. In summary, we have developed PREX-in1 and derivative small-molecule compounds that will be useful laboratory research tools for the study of P-Rex function. These compounds may also be a good starting point for the future development of more sophisticated drug-like inhibitors aimed at targeting P-Rex Rac-GEFs in cancer.

## Introduction

Rac proteins (Rac1, Rac2, Rac3 and RhoG) are small GTPases (G proteins) of the Rho family that control cytoskeletal reorganization, and thereby cell shape, adhesion, migration, phagocytosis and certain secretory processes. In addition, they control reactive oxygen species (ROS) production, gene expression, cell cycle progression and cell survival [1,2]. Rac GTPases cycle between their active (GTP-bound) and inactive (GDP-bound) conformations and can be activated by at least 20 different guanine-nucleotide exchange factors (GEFs). These Rac-GEFs are part of the larger Rho-GEF family that comprises 82 human proteins, which are either Dbl-type or DOCK-type and can activate one or more GTPases from the Rac, Cdc42, Rho and other Rho family subgroups [3,4]. GEF binding causes structural changes in Rac that result in the release of GDP and binding of free GTP, which is present at high concentrations in the cell. The GTP-bound active form of Rac interacts with effector proteins to initiate downstream cell responses.

P-Rex1 and P-Rex2 are Dbl-family GEFs that can activate Rac and Cdc42 type small GTPases *in vitro*, but only activate Rac GTPases *in vivo*, unless overexpressed [5]. P-Rex1 is abundant in leukocytes and neurons, and present at lower levels in many cell types, whereas P-Rex2 is more widely expressed but absent from leukocytes [5,6]. We first identified P-Rex1 in neutrophils, where the GEF mediates adhesion, migration, ROS production and killing of bacteria [7–13]. P-Rex1 also controls various functions of macrophages, platelets, endothelial cells, fibroblasts, melanoblasts, adipose cells and neurons that are associated with cytoskeletal dynamics, including morphology, adhesion, migration and secretory processes, as well as being involved in cell-cycle control [5,6]. The cellular functions of P-Rex2 have been studied less, but the protein is known for regulating the morphology and synaptic plasticity of cerebellar neurons, whereas P-Rex2b, a C-terminally truncated splice variant, regulates the migration of endothelial cells [5,14,15].

P-Rex Rac-GEFs are non-essential for development, but P-Rex deficient mice have distinct phenotypes. Prex1^–/–^ mice show reduced proliferation and migration of melanoblasts, which causes a developmental skin pigmentation phenotype [16,17]. They also have impaired hippocampal plasticity, which causes autism-like behaviours [18], and reduced recruitment of inflammatory cells to various organs during infection and inflammation, as well as reduced lung fibrosis under inflammatory conditions [7,11,13,19]. Prex2^–/–^ mice have impaired cerebellar plasticity and motor coordination [20,21], and show reduced glucose tolerance and altered insulin sensitivity [22]. Prex1^–/–^/ Prex2^–/–^ mice have a more severe impairment in motor control than Prex2^−/−^ [20], as well as showing immune-deficiency during bacterial pneumonia [8].

Deregulation of P-Rex Rac-GEFs is implicated in several human diseases. The *PREX1* gene maps to a locus that is linked to type 2 diabetes [23], and single-nucleotide variations in the perigenic region of *PREX1* are associated with the likelihood of obesity developing into diabetes [24]. Single-nucleotide variations and copy-number variations of *PREX1* are associated with autism spectrum disorders [18]. Importantly, both P-Rex1 and P-Rex2 are widely implicated in human cancers. P-Rex1 is overexpressed in several cancer types, including breast, prostate and melanoma, contributing to tumour initiation, growth and/or metastasis [16,25,26]. In a mouse model of melanoma driven by activated N-Ras, P-Rex1 is required for metastasis [16]. P-Rex2 is frequently mutated in human melanoma, and some of the mutations were shown to accelerate N-Ras-driven tumourigenesis in mice [27,28].

P-Rex1 and P-Rex2 are multidomain Rac-GEFs. Their N-terminal catalytic Dbl homology (DH) domain is in tandem with a membrane-targeting Pleckstrin homology (PH) domain, followed by two Dishevelled, Egl-10 and Pleckstrin (DEP) and two PSD95, Dlg1 and Zo-1 (PDZ) protein-interaction domains, and a C-terminal half with weak homology to inositol polyphosphate 4-phosphatase (IP4P) that lacks phosphatase activity [12,29]. Intramolecular interactions provide autoinhibition of the P-Rex Rac-GEF activity, meaning the basal activity of full-length P-Rex is low. Both P-Rex Rac-GEFs are directly and synergistically activated by binding the lipid second messenger phosphoinositide 3,4,5-trisphosphate (PIP_3_) which is generated by phosphoinositide 3-kinase (PI3K) and by binding the Gβγ subunits of heterotrimeric G proteins, which couple to G protein-coupled receptors [12,20]. Mutagenesis of P-Rex1 has shown that PIP_3_ binding to the PH domain and Gβγ binding to the DH domain and C-terminal portions of the protein relieve the autoinhibition, enabling Rac binding, catalytic activity, and translocation of P-Rex1 to the plasma membrane, where the GEF needs to be localized to activate Rac [5,30–33]. Another important mechanism of P-Rex1 regulation is phosphorylation by cAMP-dependent kinase (PKA), which inhibits P-Rex1 activity by stabilizing the autoinhibited conformation [34,35]. In addition, P-Rex activity is both positively and negatively modulated through phosphorylations controlled by various protein kinases and phosphatases [5,6,15], and P-Rex1 membrane localization is promoted by the GPCR-adaptor protein Norbin [36].

The crystal structure of the catalytic core of P-Rex1 has been solved in recent years. Lucato *at al*. elucidated the structure of the DHPH domain tandem in complex with constitutively active Rac1, revealing an arrangement typical for Rac-GEFs and confirming that catalytic activity is conferred by the DH domain alone [37]. A structure by Cash *et al*. of the DHPH domain tandem in complex with nucleotide-free Rac1 or Cdc42 supported these findings, showing that highly conserved regions in the P-Rex1 DH domain make extensive contacts with switch 1 and 2 of Rac1, and that residues Glu56 and Asn238 in particular are essential for Rac1 binding and catalytic activity [38]. Furthermore, a cryo-EM structure of a large portion of P-Rex1 from the second DEP domain to the C-terminus confirmed extensive autoinhibitory intramolecular interactions and their relief through allosteric changes brought about by Gβγ binding [39]. Recently, a crystal structure including the domains from the DH to the DEP1 domain and a cryo-EM structure of the full-length P-Rex1 protein have been solved by the Ellisdon lab, revealing a two-layered mechanism to relieve P-Rex1 autoinhibiton, whereby P-Rex1 binding to PIP_3_ and Gβγ causes first a rotation between the N- and C-terminal halves of the GEF and then an opening of the DH domain that enables Rac binding and catalysis [40].

With one exception, all physiological and pathophysiological roles of P-Rex family Rac-GEFs are either known or assumed to be mediated through their Rac-GEF catalytic activity. This has been established through the common usage of catalytically inactive (GEF-dead) P-Rex proteins. For example, only wild-type P-Rex1 but not the GEF-dead P-Rex1 drives prostate cancer metastasis [26], or the migration of melanoma cells [16] and fibroblasts [19], or ROS production in COS^phox^ cells [41]. The one exception is the important adaptor function of P-Rex2, but not P-Rex1, as an inhibitor of the tumour suppressor PTEN [42]. PTEN terminates PI3K signalling by metabolizing PIP_3_, and thus the inhibition of PTEN by P-Rex2, which is mediated through the PH and IP4P domains, prolongs PI3K signalling [22,42]. It has been proposed that the roles of P-Rex2 in glucose metabolism and breast cancer may depend on PTEN inhibition rather than Rac-GEF catalytic activity [22], but this remains to be investigated. Considering that the great majority of P-Rex function are mediated through the catalytic activity, it would be useful to have available P-Rex inhibitors, first-and-foremost as research tools, but potentially also as a basis for the future development of drugs in P-Rex dependent diseases.

GEFs are not generally considered good targets for pharmacological inhibition because of the relatively large surface area through which they interact with their target GTPase [43]. However, several compounds have been developed that inhibit GEFs, although mostly with limited specificity and efficacy. The exception is the naturally occurring fungal metabolite Brefeldin A (BFA), a compound which is a powerful inhibitor of Arf-GEFs and widely used in cell biology. BFA acts by stabilizing the complex between Arf-GEFs and the small GTPase Arf1, so that Arf1 cannot undergo nucleotide exchange, thereby blocking vesicle trafficking from the ER to the Golgi [43,44]. The first reported inhibitor of Rac-GEFs was the peptide W56 peptide, which mimics the surface of Rac1 that interacts with GEFs. W56 peptide was shown to block the interaction of Rac1 with the Rac-GEFs Trio-N and Tiam1 at high concentrations (above 200 µM) [45]. The most widely used Rac-GEF inhibitor is NSC23766, a small molecule developed from a computational screen for compounds that might bind to the GEF-interacting surface of Rac1. NSC23766 inhibits TrioN and Tiam1 with an IC_50_ of approximately 50 µM [46]. However, NSC23766 does not inhibit all Rac-GEFs. For example, it does not affect Vav1-dependent cell growth [46]. Derivatives of NSC23766 with lower IC_50_s have also been developed [47]. One of these, EHop-016, inhibits the interaction of Rac1 with the Tiam1 DHPH domain at similar concentrations to NSC23766, but blocks Rac-dependent cell responses such as lamellipodia formation and migration with low micromolar IC_50_, possibly through unidentified endogenous Rac-GEFs [48]. Other small-molecule Rac-GEF inhibitors include NPPD and ITX3 which inhibit Trio-N, although either with considerable cytotoxicity or limited efficacy [49,50]. EHT1864 is another compound that is widely used [51]. However, EHT1864 was shown to block nucleotide binding to Rac, and is therefore not a GEF inhibitor [52]. Most Rac-GEF inhibitors were developed for Dbl-type Rac-GEFs, but there are also compounds that inhibit DOCK-type Rac-GEFs, which have a DHR2 catalytic domain. CPYPP inhibits DOCK2-mediated Rac1 activity with an IC_50_ of 22.8 µM, and also affects other DOCK family GEFs, including DOCK1, DOCK5 and DOCK9 [53].

In summary, small-molecule inhibitors of Dbl and DOCK family Rac-GEFs have been developed by several laboratories and proven to be valuable research tools, although most of these compounds have low potency and poor selectivity for specific GEFs. Here, we describe the development and characterization of PREX-in1, a small-molecule inhibitor that targets the catalytic DH domain of the P-Rex family Rac-GEFs P-Rex1 and P-Rex2.

## Materials and methods

### Purified recombinant small GTPase and GEF proteins

All small GTPases and GEFs used were human recombinant proteins. EE-Rac1, EE-Rac2, GST-Cdc42 or GST-RhoA were expressed in Sf9 cells by baculoviral transduction and purified using their epitope tag in their post-translationally prenylated form using Triton X-114 phase separation, essentially as previously described [12,54]. Briefly, frozen pellets from a 1 litre culture of Sf9 cells transduced with high-titre baculovirus expressing the small GTPase were resuspended in ice-cold Sf9-cell lysis buffer 1 (1x PBS, 40 mM HEPES pH 7.5 at RT, 5 mM MgCl_2_, 10 mM EGTA, 300 µM GDP, 1 mM DTT, 200 µM PMSF, 20 μg/ml each of leupeptin, pepstatin A, aprotinin and antipain) and lysed by sonication. Triton X-114 was added (to 1% final concentration), and samples were ultracentrifuged at 118,000 × g for 50 min at 4°C. The supernatant was warmed at 37°C until cloudy (30–60 seconds) and centrifuged at 1300 × g for 2 min at 37°C. The upper phase containing non-lipid modified GTPase was discarded and replaced with ice-cold Sf9-cell lysis buffer. The warming/centrifugation steps were repeated twice and lipid-modified GTPase resuspended in ice-cold Sf9-cell lysis buffer 1 supplemented with Triton X-100 (1% final concentration), followed by ultracentrifugation at 118,000 × g for 50 min at 4°C. For EE-tagged GTPases, supernatants were incubated with anti-EE antibody covalently coupled to protein G-Sepharose beads (anti-EE beads, Onyx Pharmaceuticals) and incubated with end-over-end rotation for 90 min on ice. Beads were washed 3 times in wash buffer (PBS, 5 mM MgCl_2_, 1 mM DTT, 10 μM GDP, 1 mM EGTA, 0.2% Triton X-100) and 5x in elution buffer (40 mM HEPES pH 7.5, 150 mM NaCl, 5 mM MgCl_2_, 1 mM EGTA, 1 mM DTT, 10 µM GDP). Protein was eluted by three incubations with elution buffer containing peptide Ac-EEYMPME (Activotec, Comberton, UK) and cholate (first 1%, then twice 0.5%) for 20 min on ice. For GST-tagged GTPases, supernatants were incubated with glutathione Sepharose beads and were eluted with glutathione in the same manner. Proteins were concentrated in a YM-10 Centricon (Millipore) at 5000 × g, 4°C. Protein concentration and purity was determined by coomassie staining of SDS-PAGE gels. For use in the mant-GTP assay, EE-Rac 1 was expressed in BL21 (DE3) bacteria (NEB, C25271), by induction with 0.5 mM IPTG overnight at 18°C. Bacterial pellets from 500 ml cultures were resuspended in 20 ml bacterial lysis buffer (40 mM Hepes, pH 7.5 at 4°C, 150 mM NaCl, 1% Triton X-100, 5 mM MgCl_2_, 1 mM DTT, 300 μM GDP, 200 µM PMSF, and 20 μg/ml each of leupeptin, pepstatin A, aprotinin and antipain) and were lysed by sonication. 5 μg/ml DNAse and 5 μg/ml RNAse were added and the lysate incubated on ice for 10 min. Samples were ultra-centrifuged at 100,000 × g for 30 min at 4°C and EE-Rac1 isolated from the supernatant using anti-EE beads, eluted, and concentrated, as described above.

Full-length N-terminally EE-tagged P-Rex1, P-Rex2, the P-Rex1 DH and DHPH domains and the P-Rex2 DH domain were produced in Sf9 cells using baculoviral transduction and were purified using anti-EE beads, as previously described [29,30,54]. Briefly, frozen pellets from 500 ml of Sf9 culture infected with P-Rex expressing high-titre baculovirus were resuspended in ice-cold lysis Sf9-cell lysis buffer 2 (1x PBS, 1% Triton X-100, 5 mM EGTA, 1 mM EDTA, 25 mM NaF, 20 mM β-glycerophosphate, 1 mM DTT, 100 µM PMSF, 10 μg/ml each of leupeptin, pepstatin A, aprotinin and antipain) for 5 min and then ultracentrifuged at 118,000 × g for 1 h at 4°C. Supernatants were incubated with pre-washed anti-EE beads for 90 min on ice with end-over-end rotation. Beads were washed 4 times in wash buffer (2x PBS, 0.1% Triton X-100, 1 mM EGTA) and 5 times in elution buffer (1x PBS, 10% glycerol, 1 mM DTT, 1 mM EGTA, 0.01% Na azide), and protein was eluted by incubation with elution buffer containing Ac-EEYMPME peptide as described above. Protein was concentrated in a YM-100 Centricon (Millipore) at 5000 × g at 4°C. Fatty acid-free BSA and glycerol were added to final concentrations of 2 mg/ml and 50%, respectively. Protein concentration and purity were determined by coomassie staining of SDS-PAGE gels. Full-length Tiam1, expressed in and purified from Sf9 cells, was a kind gift from Angeliki Malliri, CRUK Manchester Institute, UK. The ITSN1 DHPH domains and Ect2 DHPH domains, produced in and purified from bacteria, were a kind gift from Kent Rossman, University of North Carolina, Chapel Hill, USA. The DOCK10 DHR2 domain, produced in and purified from bacteria, was a kind gift from Danni Fan, Institute of Cancer Research, London, UK. All small GTPase and GEF proteins were stored in aliquots at −80°C and were snap-frozen in liquid N_2_ after each use.

### Compounds

W56 peptide (2221), NSC23766 (2161) and EHop-016 (6248) were from Tocris Bioscience (Bristol, UK). We screed the ZINC public library of commercially available chemical compounds (http://zinc.docking.org/) for small-molecule compounds that might mimic the 16 amino acid peptide W56, which blocks Rac1 activation by Rac-GEFs [45]. We used AutoDock software (http://autodock.scripps.edu; Scripps Research Institute) to predict which of these structures might fit into the Rac binding pocket of the P-Rex1 DH domain with the best energy efficiency. We purchased the top ten compounds that emerged using this strategy from Specs (Delft, The Netherlands). Further compounds were acquired or produced on the basis of results obtained with this initial batch. Compounds #11-29 and #83-111 were from ChemBridge (San Diego, USA). Compounds #30-82 and #112-128 were synthesized using published procedures [55,56]. Identification numbers and structures of all compounds are listed in Supplemental Table 1. All compounds were dissolved in DMSO, usually as a 10 mM stock, and stored either at RT or at −20°C.

### Liposome-based GEF activity

Liposome-based GEF activity assays were carried out essentially as described [12,54], except that GEFs were incubated with inhibitor compounds prior to the assay. GEFs (full-length P-Rex1, P-Rex2, and Tiam1, P-Rex1 DH and DHPH domains, P-Rex2 DH domain, ITSN1 DHPH domains, ECT2 DHPH domains or DOCK10 DHR2 domain) were diluted in 20 mM HEPES (pH 7.0 at 4°C), 200 mM KCl, 10% ethylene glycerol, 1% betaine, 500 µM EGTA, 0.01% azide. Inhibitor compounds were added from working stocks of up to 500 μM in DMSO as a 1:20 dilution, or DMSO was added 1:20 as a vehicle control, and samples were incubated for 30 min at 30°C prior to their addition to the GEF assay reaction mix. The final 10 µl samples contained GTPase, GEF and inhibitor at concentrations as stated, in 20 mM HEPES pH 7.5, 100 mM NaCl, 1 mM MgCl_2,_ 1 mM EGTA, 1 mM DTT, 10 mg/ml fatty acid-free BSA, liposomes consisting of phosphatidylcholine, -serine and -inositol (200 µM each), with or without 10 µM PIP_3_, 1 µM GTPγS and 80 nM (1 µCi) ^35^S-GTPγS. Positive controls contained additionally 2 mM EDTA to induce maximal GTP-loading of the GTPase. Samples were incubated at 30°C for 10 min, and reactions were stopped by the addition of 400 µl ice-cold wash buffer (1x PBS, 1 mM EGTA, 10 mM MgCl_2_, 1% Triton X-100, 100 µM GTP). Small GTPases were isolated using anti-EE antibody-coupled Sepharose beads or glutathione Sepharose beads, as appropriate, by incubation on ice for 90 min with end-over-end rotation, four washes in ice-cold wash buffer and resuspension of beads in Ultima Gold scintillation fluid (Perkin Elmer). ^35^S-GTPγS incorporation was assessed by scintillation counting (Packard 1600TR, Liquid Scintillation Analyser).

### Mant-GTP GEF activity

The mant-GTP GEF activity assay measures the binding of fluorescent 2’/3’-O-(N-Methyl-anthraniloyl)-guanosine-5’-triphosphate (mant)-GTP to Rac, making use of the spectroscopic differences between bound and free mant-GTP. P-Rex1 DHPH domain protein was diluted to 375 nM in exchange buffer (20 mM Tris, pH 7.5 RT, 50 mM NaCl, 200 mM KCl, 4 mM MgCl_2,_ 50 μg/ml fatty-acid-free BSA). The inhibitors PREX-in1 or EHop-016 were diluted to various concentrations in exchange buffer containing 15% DMSO. 4.4 µl P-Rex1 DHPH domain protein were incubated with 1.1 µl PREX-in1 or EHop-016 for 30 min at 30°C, or were mock treated with 1.1 µl vehicle (15% DMSO). 5 μl of the P-Rex1 DHPH / inhibitor mix was then added to 10 µl of a 1.5x reaction mix containing 225 nM bacteria-derived EE-Rac1, 7.5 µM mant-GTP and 15 µM GTP in exchange buffer in a 96-well plate. Final assay concentrations were 150 nM EE-Rac1, 100 nM P-Rex1 DHPH domain, 5 μM mant-GTP, 10 µM GTP, 10 mg/ml fatty-acid-free BSA, 1% DMSO, and the indicated concentrations of inhibitor. Mant-GTP fluorescence was measured in real time in a PHERAstar 384-well fluorescence plate reader at 25°C for 40 min using 360 nm excitation and 440 nm emission.

### Cell cycle

Human embryonic kidney 293 (HEK-293) cells were grown at 37°C in a humidified 5% CO_2_ atmosphere in Dulbecco’s modified Eagle medium (DMEM, Gibco) supplemented with 10% foetal bovine serum (FBS), 100 units/ml penicillin and 100 µg/ml streptomycin. Cells were passaged by tryptic digest 1:4 approximately every 2 days. To measure cell cycle stages, 2 ml HEK293 cells were plated per well of a 6-well dish (Thermo Scientific, Nunc) at 10^5^ cells/ml. After 24 h, the medium was replaced with fresh medium containing various concentrations of PREX-in1, as indicated, or 10 μM NSC23766, or vehicle control (0.1% DMSO), and cells were incubated for a further 24 h. The medium was removed but kept aside to recover any detached cells. Wells were washed with PBS, and cells recovered by trypsinization. Cell suspensions were pooled with the culture supernatant and centrifuged at 433 × g for 5 min at RT. Cells were washed in PBS, resuspended in ice-cold 70% ethanol/PBS, and incubated for 30 min at 4°C for fixation and permeabilization. Cells were washed once more in PBS, then stained with 50 µg/ml propidium iodide in PBS containing 100 µg/ml RNase for 30 min at 37°C. Cells were analysed by flow cytometry for the intensity of propidium iodide staining using a FACSCalibur flow cytometer. 10,000 cells were analysed per sample. Data analysis was done using FloJo software.

### Cell morphology

Pig aortic endothelial (PAE) cells, which stably overexpress PDGF receptor beta [57], were grown at 37°C in a humidified 5% CO_2_ atmosphere in Ham’s F-12 medium with L-glutamine (PAA) supplemented with 10% FBS, 100 units/ml penicillin and 100 µg/ml streptomycin (complete medium). Cells were passaged by tryptic digest 1:4 approximately every 2 days. PAE cells were transfected with eGFP-P-Rex1 by electroporation as described [30], resuspended in complete medium without antibiotics and plated onto sterile 22 mm glass coverslips in 6-well plates. After 12 h, cells were washed twice in Ham’s F-12 and serum starved in Ham’s F-12 containing 1% fatty acid-free BSA) for 8 h. PREX-in1 was added to the medium to a concentration of 1 µM, or cells were mock-treated with vehicle control (0.05% DMSO), for 15 min at 37°C. Cells were stimulated with 10 ng/ml PDGF for 10 min, or were mock-stimulated, and then fixed in 4% paraformaldehyde, 50 mM Pipes pH 6.8, 1 mM EGTA, 1 mM MgCl_2_ for 15 min at RT. Cells were washed in PBS, permeabilized for 10 min in PBS containing 0.2% Triton X-100, washed twice in PBS and once in PBS, 0.1% BSA. The F-actin cytoskeleton was stained by incubating samples with TRITC-Phalloidin (Sigma P1951) in PBS, 0.1% BSA for 45 min at RT in the dark. Coverslips were washed twice in PBS, rinsed once in water, and mounted onto slides using Aqua Polymount (Polysciences, Inc.). Samples were imaged using an AxioImager D2 fluorescence microscope (Zeiss, ×200 magnification), taking 15–20 representative images for each duplicate coverslip. Images were analysed blinded to experimental design using ImageJ, for both manual and automated analysis. Using manual analysis, each cell was assigned to a morphology category (basal, with lamellipodia and membrane ruffles, spread with ruffles). With automated analysis, the surface area and circularity of all cells were quantified using the ‘Set Measurements’ analysis tools of ImageJ after assigning a mask to each cell. To determine the proportion of spread cells, cells with a surface area more than 50% larger than the mean under basal conditions (1246 μm^2^) were considered spread. eGFP-P-Rex1 expressing cells were compared to non-green fluorescent cells on the same coverslip.

### Mice

*Prex^−/−^* mice (*Prex1^−/−^Prex2^−/−^*) with general P-Rex deficiency were described previously [8,9,20,58] and were compared to *Prex^+/+^ (Prex1^+/+^Prex2^+/+^*) control mice on the same genetic background (C57Bl6/129Ola backcrossed seven times to C57Bl6). Mice were group-housed (up to 5) under specific opportunistic pathogen-free isolator conditions in Babraham’s small animal barrier facility that uses 12 h light/dark cycles with dusk and dawn settings, and were fed chow diet and water *ad libitum*. Mice from both sexes were used for experiments, at young-adult age (between 8 and 14 weeks), and were sex- and age-matched between genotypes within experiments. Animal breeding and experiments were carried out with approval from the local Animal Welfare Ethical Review Body under the British Home Office Animal Scientific Procedures Act 1986.

### Neutrophil ROS production

Mature primary neutrophils were isolated from the bone marrow of age- and sex-matched *Prex^+/+^ and Prex^−/−^* mice freshly each day at 4°C, using Percoll^Plus^ (GE Healthcare) gradient, as described, using endotoxin-free media throughout [10]. Isolated neutrophils were 70–90% pure, as assessed by KwikDiff staining of cytospins. ROS production was measured by real-time chemiluminescence assay as described [9], except that neutrophils were incubated with PREX-in1 before the assay. Briefly, isolated neutrophils were resuspended at 8.33 × 10^6^ cells/ml in Dulbecco’s Phosphate-Buffered Saline containing Ca^2+^ and Mg^2+^ (Sigma, D8662), supplemented with 0.1% glucose and 4 mM NaHCO_3_ (DPBS^++++^). 0.3 µl of 2 mM PREX-in1 in DMSO was added to 180 µl neutrophils, and samples were incubated for 5 min at 37°C, or were mock treated with vehicle (DMSO) control. 180 µl of prewarmed DPBS^++++^ containing 16 units/ml horseradish peroxidase (Sigma, P8375) and 120 µM luminol (Sigma-Aldrich, 123,072) were added, and samples were incubated for a further 2 min. Horseradish peroxidase and luminol enable measurement of extra- and intracellular ROS combined. 150 μl of this mix was pipetted into a well of a prewarmed 96-well plate. 100 µl of prewarmed 25 µM fMLP or 1.25 µM PMA (in DPBS^++++^) were added to stimulate the ROS response, either using an automated injection port (fMLP) or manually (PMA). Real-time ROS production was recorded in a Berthold MicroLumat Plus luminometer (Berthold Technologies) at 37°C, for 2.5 min with fMLP stimulation or for 10 min with PMA stimulation. Final assay concentrations were 2.5 × 10^6^ neutrophils/ml, 1 µM PREX-in1, 0.075% DMSO, and 10 µM fMLP or 500 nM PMA. The luminometer software and Microsoft Excel were used to calculate the area under the curve of ROS production, integrated over 2 min for the fMLP response and 10 min for the PMA response, and to determine the height and timing of the peak of ROS production.

### Data analysis

Sample size and numbers of independent experiments are detailed in figure legends. Excel 2016 and GraphPad Prism 9.0 were used for tabulation, statistical analysis and plotting graphs. Data were tested for normality of distribution using Shapiro–Wilk test to determine if parametric or non-parametric statistical analysis was required. For ImageJ cell morphology data, outliers were identified using the default ROUT setting of GraphPad Prism (Q = 1%) and removed prior to analysis. For comparisons between two groups, Student’s t-test was used. For all other data, one-way, two-way, or three-way ANOVA were used, as appropriate. Effect size and variance are reported as group mean ± standard error. P-values reported are from multiplicity-adjusted post-hoc comparisons, as detailed in the figure legends. The threshold for statistical significance was set at p < 0.05. Only statistically significant comparisons are denoted in the figures.

## Results

### PREX-in1 is a small-molecule inhibitor of P-Rex1 Rac-GEF activity

NSC23766 is a widely used compound that inhibits the activation of Rac by the Rac-GEFs Trio-N and Tiam1 [46]. To begin our search for P-Rex inhibitors, we performed pilot experiments testing the ability of NSC23766 to inhibit the PIP_3_-stimulated Rac-GEF activity of full-length P-Rex1 using an established liposome-based *in vitro* GEF activity assay [12,54]. NSC23766 did not appear to inhibit P-Rex1 **(Supplemental Figure 1A)**. In contrast, a pilot experiment with the 16 amino-acid peptide W56, which mimics the Rac-GEF binding surface of Rac1 and reduces the interaction of Rac1 with Trio-N and Tiam1 [45], did appear to inhibit the Rac-GEF activity of P-Rex1 in a dose-dependent manner **(Supplemental Figure 1B)**. The efficacy of W56 peptide in these pilot experiments was poor, its IC_50_ was ~250 µM, although comparable to its reported effects on Trio-N and Tiam1 [45]. Given the low efficacy and lack of selectivity, W56 peptide did not meet our criteria for a P-Rex1 inhibitor. Instead, we used W56 peptide as a starting point for developing small-molecule P-Rex inhibitors.

**Figure 1.**
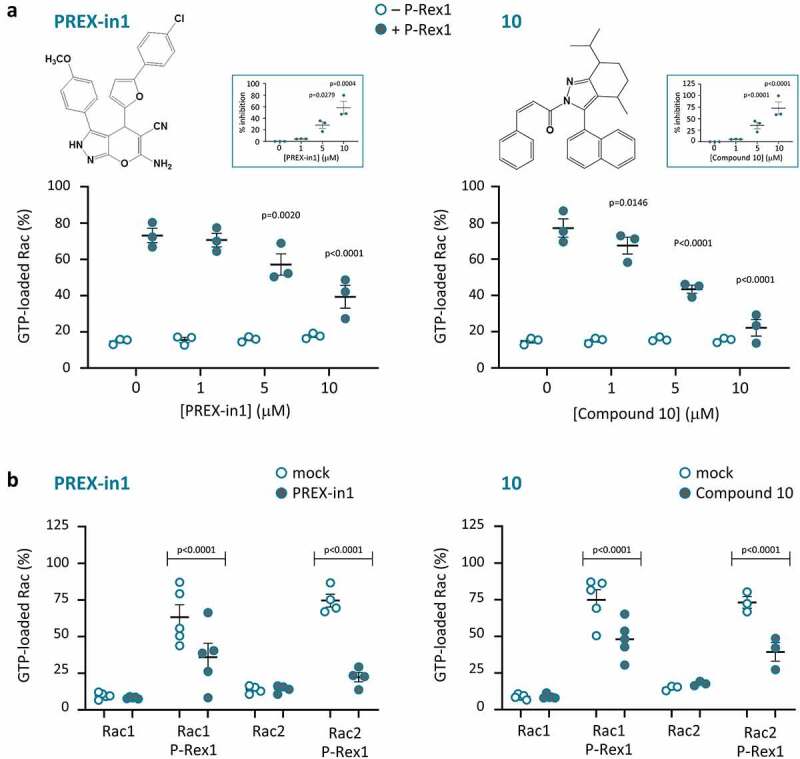
P-Rex1 Rac-GEF activity is inhibited by small molecules PREX-in1 and compound 10. (*a*) Full-length recombinant EE-P-Rex1 (50 nM final concentration, filled symbols), or control samples without P-Rex1 (open symbols), were incubated with the indicated concentrations of PREX-in1 (left-hand panels) or compound 10 (right-hand panels) for 30 min, before Rac-GEF activity was measured by liposome-based GEF assay in the presence of liposomes that contained 10 µM PIP_3_ to activate P-Rex1, using prenylated GDP-loaded EE-Rac2 as substrate (100 nM final). Rac2 activity (GTP-loading) is expressed as % of maximal GTP loading in the EDTA positive control. Inserts: inhibition of P-Rex1 is plotted using Rac-GEF activity without compound as 0% and Rac2 GTP-loading without P-Rex1 as 100%. Data are mean ± SEM of duplicate measurements from 3 independent experiments for each compound. Statistics are two-way ANOVA in main panels and one-way ANOVA in inserts, both with Dunnett’s multiple comparisons test. *(b)* The Rac-GEF activity of full-length recombinant EE-P-Rex1 was measured as in *(a)* after incubating samples with or without P-Rex1 in the presence of 10 µM PREX-in1 or 10 µM compound 10 (closed symbols), or after mock-treatment (open symbols), by liposome-based GEF assay with liposomes containing PIP_3_, and comparing prenylated EE-Rac1 and EE-Rac2 as substrates, as indicated. Rac GTP-loading is expressed as % of the EDTA positive control. Data are mean ± SEM of duplicate measurements from 3–5 independent experiments for each compound and Rac GTPase; each dot is the mean of one experiment. Statistics are two-way ANOVA with Sidak’s multiple comparisons test.

We screened the ZINC public library of commercially available chemical compounds for small molecules that mimic the W56 peptide. We subjected the top 1000 hits from this screen to analysis by AutoDock software to predict which structures might fit into the Rac binding pocket of the catalytic DH domain of P-Rex1 with the best energy efficiency. As a crystal structure of the P-Rex1 DH domain was not available at that stage, we employed for this docking analysis a homology model constructed from the crystal structure of the Tiam1 DH domain in complex with Rac1 [59]. We purchased the top ten compounds that emerged from this strategy. Of these 10 compounds, seven were soluble in DMSO, and were tested in the GEF activity assay at concentrations of 1, 5 and 10 µM. Two of these compounds (#1 and #10) inhibited the PIP_3_-stimulated Rac-GEF activity of full-length P-Rex1 within that concentration range (Figure 1a). Both compounds inhibited P-Rex1 when either Rac1 or Rac2 were used as the substrate. In contrast, neither compound affected the basal GTP-loading of Rac1 or Rac2 in the absence of P-Rex1 (Figure 1b), nor the GEF-independent maximal GTP-loading of the GTPases achieved by incubation with EDTA (data not shown), suggesting that the compounds worked through P-Rex1 rather than through the Rac GTPases. We decided to pursue compound #1 rather than #10, because #1 was less hydrophobic (LogP 4.81 compared to 7.61), and its structure showed greater potential for the synthesis of analogues. We named compound #1 ‘PREX-in1’, for P-Rex inhibitor 1.

### PREX-in1 inhibits P-Rex1 and P-Rex2 through their catalytic DH domain

**Figure 2.**
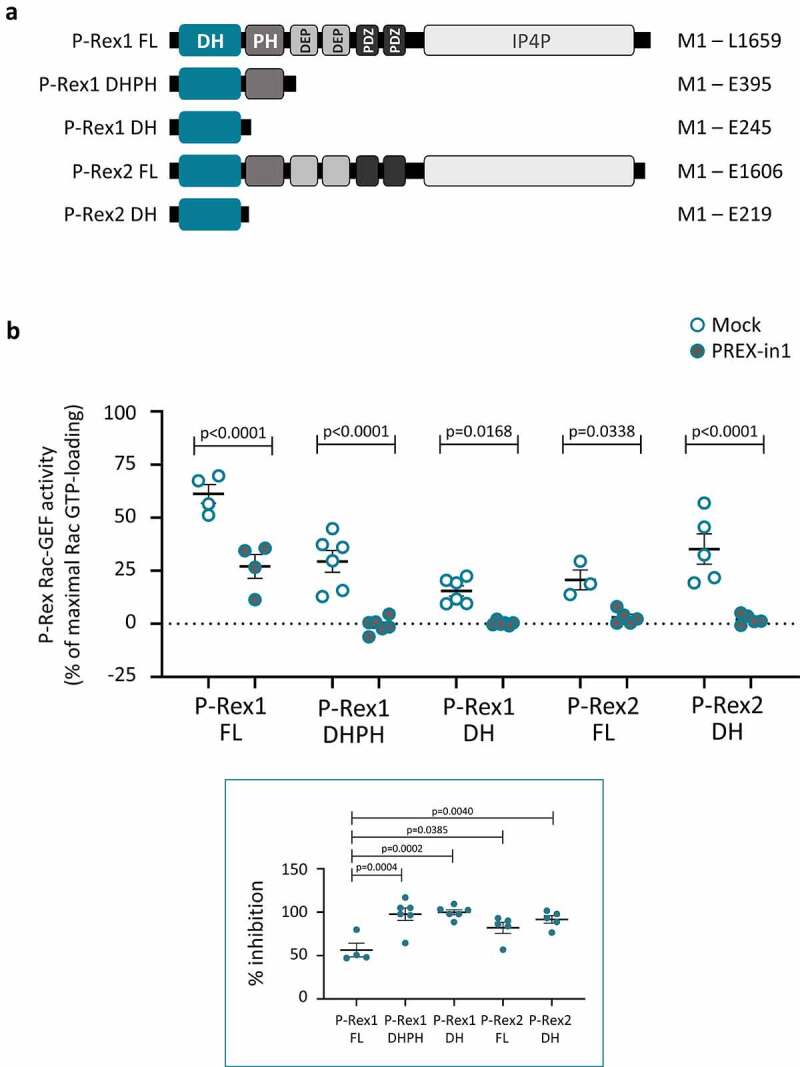
PREX-in1 inhibits P-Rex1 and P-Rex2 through the catalytic DH domain. *(a)* Schematic showing the domain structures of full-length (FL) P-Rex1 and P-Rex2 and the isolated P-Rex1 DHPH and DH domain and P-Rex2 DH domain proteins. *(b)* Upper panel: Full-length recombinant EE-P-Rex1 (50 nM) or P-Rex2 (40 nM), or the isolated DH domains (200 nM) or DHPH domains (100 nM), were incubated with 10 M PREX-in1 for 30 min (filled symbols), or were mock-treated (open symbols). Their Rac-GEF activity was then measured by liposome-based GEF assay using 100 nM prenylated Rac1 as substrate, in the presence of liposomes with 10 µM PIP_3_ for the full-length GEF protein, or without PIP_3_ for the isolated domains. Rac-GEF activity is plotted as Rac GTP-loading in the presence of GEF, expressed as % of maximal Rac GTP-loading in the EDTA positive control. Lower panel: inhibition of P-Rex is plotted using Rac-GEF activity without PREX-in1 as 0% and basal Rac1 GTP-loading without P-Rex as 100%. Data are mean ± SEM of 4–6 independent experiments for each P-Rex protein; each dot is the mean of one experiment. Statistics are two-way ANOVA for the main panel and one-way ANOVA for the insert, both with Sidak’s multiple comparisons test.

To examine if PREX-in1 inhibits P-Rex1 through the catalytic DH domain, as designed, we compared its effects on the PIP_3_-stimulated Rac-GEF activity of full-length P-Rex1 and on the constitutively active isolated P-Rex1 DH and DHPH domains (domain architecture is shown in Figure 2a). The PIP_3_-stimulated Rac-GEF activity of full-length P-Rex1 was reduced by 56%, and the constitutive Rac-GEF activity of the isolated DH and DHPH domains was inhibited by 98% and 100%, respectively, in the presence of 10 µM PREX-in1 (Figure 2b). Hence, we concluded that PREX-in1 targets the catalytic DH domain of P-Rex1. The increased efficacy of PREX-in1 with the isolated domains compared to the full-length protein suggested that the compound may have increased access to the catalytic domain in the absence of the auto-inhibitory intramolecular interactions provided by the other P-Rex1 domains.

Next, we asked whether PREX-in1 could inhibit P-Rex2 as well as P-Rex1. P-Rex1 and P-Rex2 Rac-GEFs have an identical domain structure (Figure 2a). We expected that the compound would work on both P-Rex family GEFs, as the primary sequences of the full-length proteins are 57% identical/74% similar, and their catalytic DH domains are 71% identical/83% similar [20]. Indeed, 10 µM PREX-in1 inhibited both the PIP_3_-stimulated Rac-GEF activity of full-length P-Rex2 (by 82%) and the constitutive Rac-GEF activity of the isolated P-Rex2 DH domain (by 92%) (Figure 2b). In conclusion, PREX-in1 inhibits the Rac-GEF activities of both P-Rex1 and P-Rex2 through their catalytic DH domain.

### PREX-in1 also inhibits P-Rex1 in another type of GEF activity assay, whereas EHop-016 has no effect

**Figure 3.**
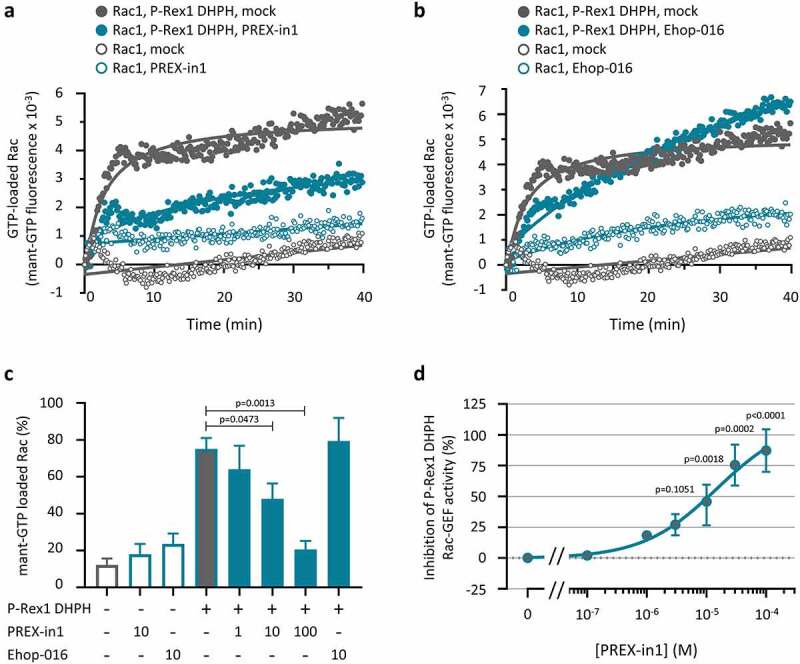
PREX-in1 (but not EHop-016) inhibits the P-Rex1 DHPH domain in a second type of GEF assay. *(a, b)* Recombinant EE-P-Rex1 DHPH domains (100 nM, filled symbols), or control samples without P-Rex1 (open symbols) were incubated with 10 µM PREX-in1 *(a)* or EHop-016 *(b)* for 30 min (green symbols), or were mock-treated (grey symbols), before their Rac-GEF activity was measured in real time by mant-GTP GEF assay using 150 nM EE-Rac1 produced in bacteria as substrate. Rac1 activity (GTP-loading) is expressed as mant fluorescence. *(c)* Quantification of P-Rex1 DHPH Rac-GEF activity from experiments such as those shown in *(a, b)* under the indicated conditions (inhibitor concentrations are in µM), expressed as area under the curve (AUC) integrated over 32 min. Data are mean ± SEM of 4 independent experiments. Statistics are one-way ANOVA with Sidak’s multiple comparisons test. *(d)* Dose response of PREX-in1 dependent inhibition of P-Rex1 DHPH Rac-GEF activity in the mant-GTP assay as in *(a,b)*, using the indicated concentrations of PREX-in1, quantified by integration of the AUC as in *(c)*. Data are mean ± SEM of 2–4 independent experiments. Statistics are mixed-effects one-way ANOVA with Holm-Sidak’s multiple comparisons test.

To ascertain if PREX-in1 can inhibit P-Rex1 independently of the liposomes provided in the previous experiments, we tested its effect on the P-Rex1 DHPH domain in a second type of GEF activity assay, which measures loading of bacterially derived EE-Rac1 with the fluorescent analogue mant-GTP in real time. We compared PREX-in1 with another Rac-GEF inhibitor, EHop-016, which is known to block the interaction of Rac1 with Tiam1 [48]. Similar to the liposome-based assay, 10 µM PREX-in1 inhibited the P-Rex1 DHPH-dependent mant-GTP loading of Rac1, without reducing the basal mant-GTP loading of Rac1 in the absence of P-Rex1 (Figure 3a). In contrast, 10 µM EHop-016 did not affect the P-Rex1 dependent mant-GTP loading of Rac1 (Figure 3b), suggesting that EHop-016 is not a direct P-Rex inhibitor. Dose response experiments showed that PREX-in1 was somewhat less efficient at inhibiting P-Rex1 DHPH in the mant-GTP assay than the liposome-based assay, with an IC_50_ of around 10 µM (Figure 3c, d). Overall, however, these data showed that PREX-in1 is a P-Rex inhibitor regardless of the type of GEF activity assay used.

### PREX-in1 inhibits P-Rex Rac-GEFs, but not a selection of other Rho-GEFs

**Figure 4.**
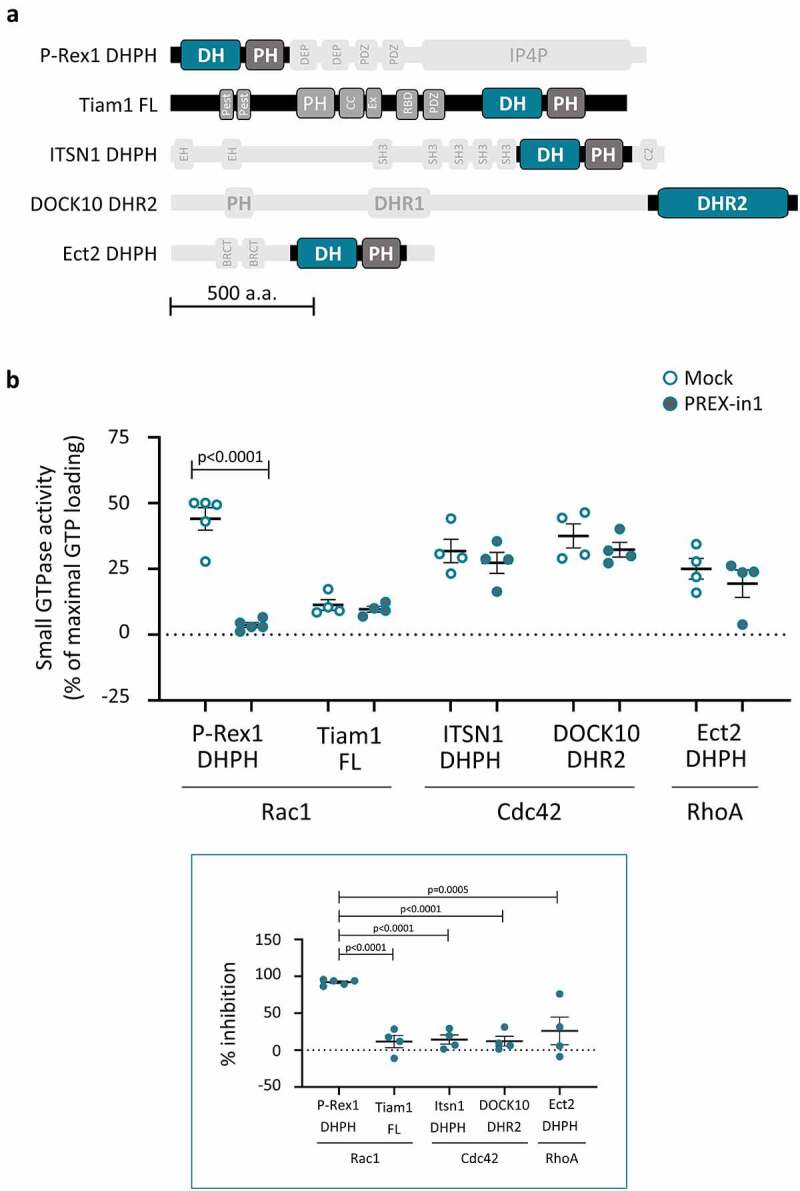
PREX-in1 inhibits P-Rex Rac-GEFs but not a selection of other Rho-GEFs. *(a)* Schematic of the domain structure of the Rho-GEFs tested, with domains used in GEF activity assays highlighted. *(b)* Upper panel: Recombinant Rho-GEFs or catalytic domains (100 nM P-Rex1 DHPH, 50 nM full-length Tiam1, 100 nM ITSN1 DHPH, 765 nM DOCK10 DHR2, or 500 nM ECT2 DHPH) were incubated with 10 µM PREX-in1 for 30 min (filled symbols), or were mock-treated (open symbols). Their GEF activity was then measured by liposome-based GEF assay using their respective Sf9-cell derived prenylated target Rho-GTPase (200 nM) as substrate. Rho-GTPase activity is expressed as % of maximal GTP loading in their respective EDTA positive controls. Lower panel: inhibition of Rho-GEFs by PREX-in1 is plotted using GEF activity without PREX-in1 as 0%, and basal GTP-loading of the target Rho-GTPase (without GEF) as 100%. Data are mean ± SEM of 4–5 independent experiments for each Rho-GEF protein; each dot is the mean of one experiment. Statistics are two-way (upper panel) and one-way (lower panel) ANOVA, both with Sidak’s multiple comparisons test.

We examined whether PREX-in1 has any selectivity for P-Rex family Rac-GEFs over other types of Rho-GEFs, by comparing the effects of 10 µM PREX-in1 on the GEF activity of P-Rex1 DHPH protein and a panel of other Dbl-type and DOCK-type Rho-GEFs using the liposome-based GEF activity assay. The panel of Rho-GEFs included the Rac-GEF Tiam1 (full-length), the Cdc42-GEF ITSN1 (DHPH) and the RhoA-GEF Ect2 (DHPH), as well as the DOCK-type Cdc42-GEF DOCK10 (DHR2); domain structures are shown in Figure 4a. Each of these GEFs was tested with its respective target GTPase. In contrast to P-Rex1 DHPH Rac-GEF activity, which was inhibited by >90% as expected, none of the other GEFs were affected by 10 µM PREX-in1 (Figure 4b). This suggests that PREX-in1 has selectivity for P-Rex Rac-GEFs over a range of other Rho-GEFs.

### Structure activity relationship tests reveal the important structural features of PREX-in1 and its derivatives

**Figure 5.**
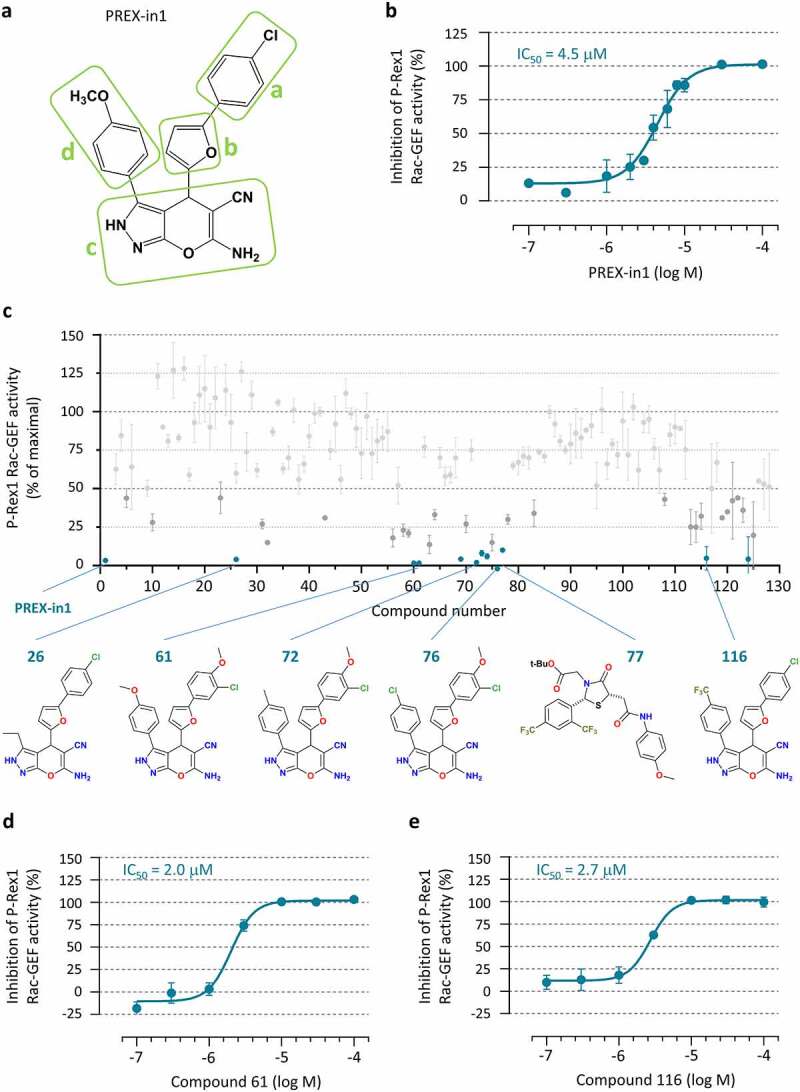
Structure activity relationship of PREX-in1 and its derivatives. *(a)* Chemical structure of PREX-in1, nominally divided into four regions, containing the (a) chlorophenyl, (b) furan, (c) 6-amino-2,4-dihydropyrano[2,3-c]pyrazole-5-carbonitrile and (d) methoxy-benzene groups. *(b)* Dose response of the inhibition of recombinant EE-P-Rex1 DHPH domains by PREX-in1 in the liposome-based GEF assay with 200 nM prenylated EE-Rac2 as substrate. Inhibition of P-Rex DHPH is plotted using Rac-GEF activity without PREX-in1 as 0%, and Rac GTP-loading of Rac without P-Rex1 DHPH as 100%. Data are mean ± SEM of 5 independent experiments. The IC_50_ was determined by application of a non-linear fit curve. *(c)* Structure activity relationship tests measuring the effect of PREX-in1 derivatives on P-Rex1 Rac-GEF activity. Compounds (10 µM) were incubated for 30 min with 200 nM iDH EE-P-Rex1 DH domain (compounds 11–29) or with 100 nM EE-P-Rex1 DHPH domains (PREX-in1, compounds 2–10 and 30–128) before being tested in the liposome-based GEF activity assay with 200 nM prenylated EE-Rac1 as substrate. Rac-GEF activity is plotted with the activity in the absence of compound set to 100% and basal Rac1 GTP-loading (without P-Rex1) set to 0%. Data are mean ± SEM from ≥ 3 independent experiments; dark grey dots denote compounds that inhibit P-Rex1 > 50%, and dark green dots are compounds that inhibit >90%. Structures of representative compounds are shown. *(d, e)* Dose responses of the inhibition of recombinant EE-P-Rex1 DHPH domains by compound 16 *(d)* or compound 116 *(e)* were assayed as in *(b)*. Data in *(d)* are mean ± range of 2 experiments representative of 4 performed under similar conditions. Data in *(e)* are mean ± SEM of 3 independent experiments. The IC_50_s were determined by application of a non-linear fit curve.

We used structure activity relationship (SAR) testing to investigate the essential features of PREX-in1 for inhibiting P-Rex, with the aim of developing inhibitors with even better efficacy. The PREX-in1 compound is a 6-amino-4-[5-(4-chlorophenyl)-2-furyl]-3-(4-methoxyphenyl)-2,4-dihydropyrano[2,3-c]pyrazole-5-carbonitrile. To investigate which parts are required for P-Rex inhibition, we notionally separated the PREX-in1 molecule into four different regions, containing (a) the chlorophenyl group, (b) the furan ring, (c) the 6-amino-2,4-dihydropyrano[2,3-c]pyrazole-5-carbonitrile group, and (d) the methoxybenzene (Figure 5a). The IC_50_ of PREX-in1 mediated inhibition of the P-Rex1 DHPH Rac-GEF activity was determined to be 4.5 µM and set as the benchmark (Figure 5b). We purchased or synthesized 118 PREX-in1 analogues (#11-#128) made up broadly of two different classes, in several iterative rounds of development. The chemical structures of these compounds are shown in **Supplemental Table 1**. Class I compounds (#11-#62, #69-#76, and #112-#117) had variations in regions (a), (b) or (d), alone or in combination, whereas region (c) was largely retained as a backbone. Class II compounds (#63-#68, #77-#111, and #118-#128) did not contain region (c), but were predicted by molecular modelling to occupy a similar 3D space as PREX-in1. All analogues were screened for their ability to inhibit the constitutively active P-Rex1 DH or DHPH domains at a concentration of 10 µM in the liposome-based GEF activity assay. Among Class I analogues, seven compounds reduced P-Rex1 Rac-GEF activity to below 10% (#26, 61, 72, 73, 74, 76 and 116) (Figure 4c). These inhibitors had a similar structure to PREX-in1, with alterations to region (a) in compound #61, region (d) in compound #26, or both in compounds #72-74 and #76, while compound #116 had additional alterations in region (c) (Figure 4c **and Supplemental Table 1)**. Importantly, the SAR testing indicated which features of PREX-in1 are important for inhibition. For example, removing the chlorine atom from region (a), or any major alterations to this region were unfavourable, as were any alterations to region (b). Class II analogues were generally less efficient, with the exception of compound #77, which inhibited P-Rex1 activity by 90% at 10 µM. Dose response curves of the most efficient compounds showed that compounds #61 and 116 were the best P-Rex1 inhibitors, with IC_50_s of 2.0 and 2.7 µM, respectively (Figure 5 d and e). Hence, SAR testing allowed us to develop PREX-in1 analogues with twofold improved efficacy, making these compounds some of the most efficient Rho-GEF inhibitors described to date, as well as helping understand the structural requirements for P-Rex inhibition.

### Long-term PREX-in1 treatment induces cell death in HEK-293 cells

**Figure 6.**
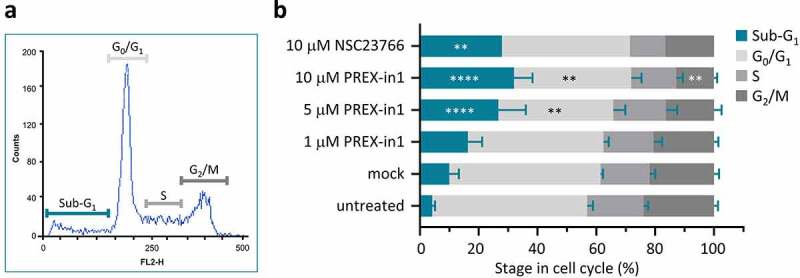
PREX-in1 affects the cell cycle in a similar manner to NSC23766. (*a*) Representative flow cytometry histogram showing cell-cycle stages of propidium iodide stained HEK293 cells after mock-treatment with vehicle (DMSO) for 24 h. (*b*) Quantification of cell-cycle stages of HEK293 cells determined as in (a), after 24 h treatment with increasing concentrations of PREX-in1, as indicated, or with 10 µM NSC23766. Data are mean % ± SEM of 5 experiments performed in triplicate. Statistics are two-way ANOVA with Dunnett’s multiple comparisons tests, comparing each cell-cycle stage to the mock-treated condition.

Next, we moved to testing the effects of PREX-in1 on cells. Rho-GEF inhibitors often exhibit considerable cytotoxicity due to off-target effects. To assess the effects of PREX-in1 on cell survival, we measured the cell cycle stages of HEK-293 cells using propidium iodide staining after treatment with increasing doses of PREX-in1 for 24 h, compared to treatment with 10 µM NSC23766. Increasing doses of PREX-in1 raised the proportion of cells in the sub-G_1_ phase, which are dead cells, with a concomitant decrease in all other cell cycle phases, particularly G_0_/G_1_ and G_2_/M (Figure 6). Hence, PREX-in1 promotes cell death. As endogenous P-Rex1 expression in HEK-293 cells is low [36], this is most likely an off-target cytotoxic effect of PREX-in1. However, the level of cell death induced by PREX-in1 at 10 µM was similar to that of NSC23766 at the same concentration (Figure 6b). Overall, therefore, it appears that long-term treatment with PREX-in1 induces similar levels of cytotoxicity as NSC23766. In further cell-based assays, we therefore treated cells with the lowest effective concentration of PREX-in1 and for the shortest effective time.

### PREX-in1 inhibits the P-Rex1-dependent spreading of endothelial cells in response to PDGF stimulation

**Figure 7.**
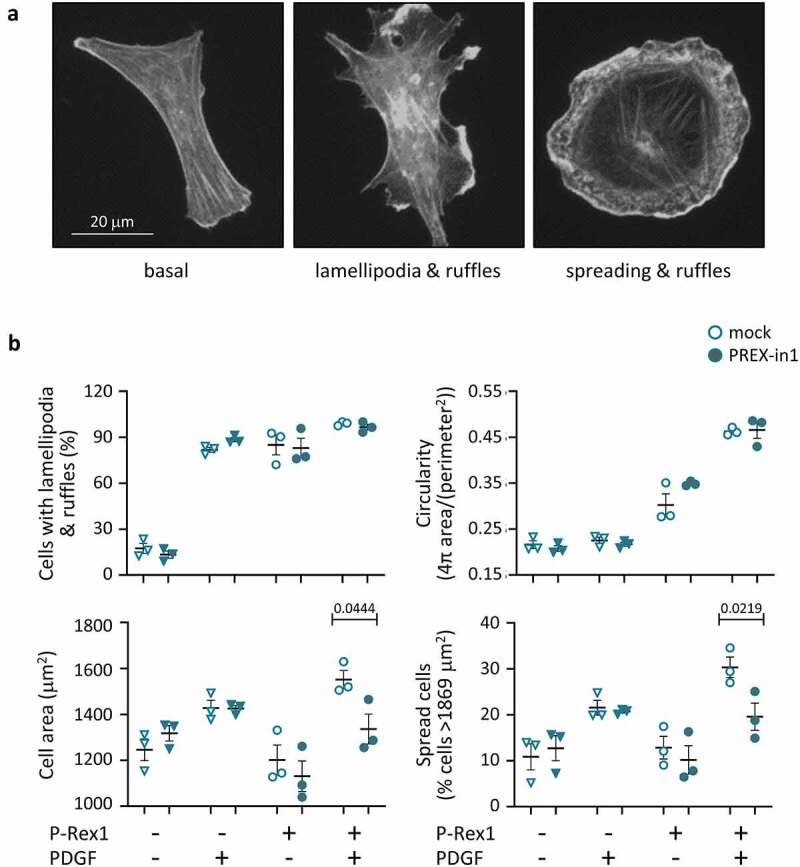
PREX-in1 inhibits the P-Rex1-dependent spreading of endothelial cells upon stimulation with PDGF. PAE cells were transfected with eGFP-P-Rex1, or mock-transfected, serum-starved, and then treated with 1 µM PREX-in1 for 15 min (filled symbols), or mock-treated (open symbols), followed by stimulation with 10 ng/ml PDGF for 10 min, or mock-stimulation, as indicated. Cells were fixed, permeabilised and stained with TRITC-phalloidin. Images were acquired by wide-field fluorescence microscopy and analysed blinded to experimental design for both manual and automated ImageJ analysis. (*a*) Representative fluorescence micrographs of the F-actin structure (phalloidin stain) of PAE cells expressing eGFP-P-Rex1 (right) or not (left and middle), and stimulated with PDGF (middle and right) or not (left). *(b)* Quantification. Cell morphologies (top left panel) were categorized manually according to the presence of lamellipodia, membrane ruffles and spread phenotype. Circularity (top right) and cell surface area (bottom left) were quantified using automated ImageJ analysis. The proportion of spread cells (bottom right) was defined as cells with a minimum surface area of 1869 m^2^, >50% larger than the mean under basal conditions (1246 μm^2^). Data are mean ± SEM from 3 independent experiments. At least 78 cells were analysed per condition for each experiment. Statistics are three-way ANOVA with Sidak’s multiple comparisons test.

To determine if PREX-in1 has on-target effects in cells, we tested endothelial cell spreading. Stimulation of PAE cells with PDGF results in a PIP_3_-dependent increase in Rac activity, leading to membrane ruffling and lamellipodia formation. Overexpression of full-length P-Rex1 causes similar morphologies, and combined with PDGF stimulation results in cell spreading, giving cells the characteristic ‘fried egg’ shape induced by constitutively active Rac1 [12] (Figure 7a). We tested the effects of PREX-in1 on these P-Rex1 dependent cell morphologies.

We transfected PAE cells with full-length eGFP-P-Rex1, serum-starved them, and treated them with 1 µM PREX-in1 for 15 min (or mock-treated with vehicle) prior to stimulation with PDGF, or mock-stimulation. Images were evaluated in a blinded manner both manually and by automated ImageJ analysis. Expression of eGFP-P-Rex1-induced lamellipodia formation, membrane ruffling and cell spreading, particularly in combination with PDGF stimulation, as expected (Figure 7b). Manual analysis suggested that PREX-in1 did not affect the proportion of cells with lamellipodia or membrane ruffles, and this was supported by the lack of effect of PREX-in1 on cell circularity determined by automated analysis. However, PREX-in1 did inhibit the P-Rex1 dependent spreading of PDGF-stimulated cells. It reduced both mean cell surface area and the proportion of spread cells (>50% larger than the average under basal conditions) (Figure 7b). PREX-in1 did not affect the morphology of mock-transfected cells, confirming that the inhibitor did not cause any noticeable cytotoxicity under these conditions, and that it exerted its effects on cell morphology through P-Rex1.

### PREX-in1 inhibits the fMLP-stimulated production of ROS in primary mouse neutrophils

**Figure 8.**
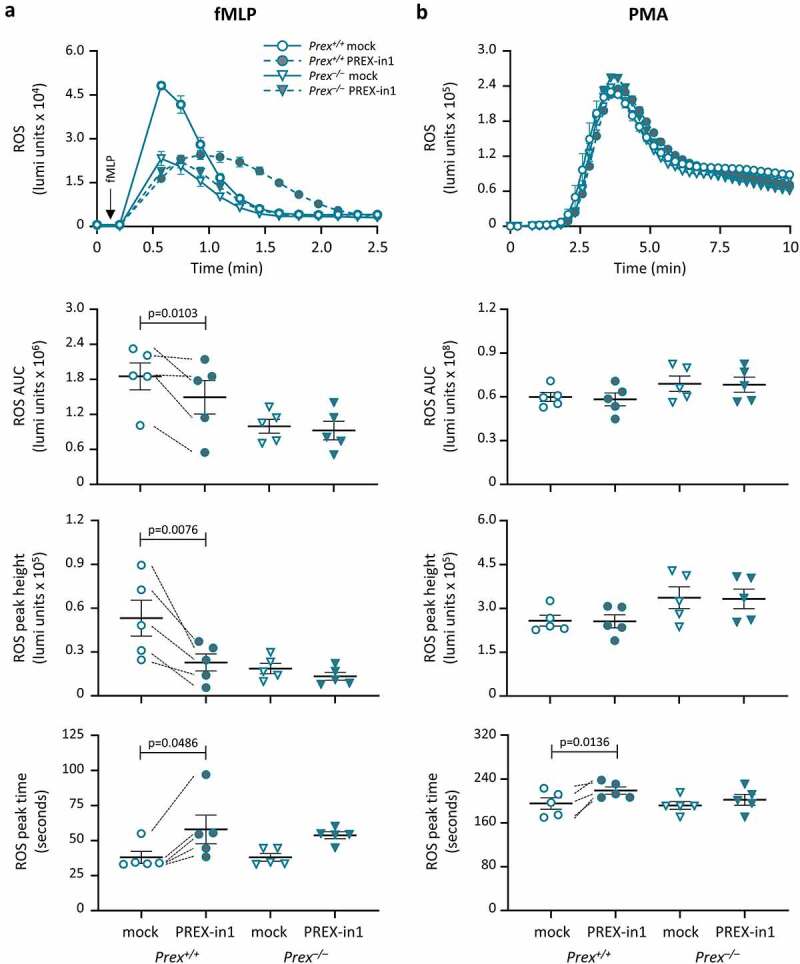
PREX-in1 inhibits the P-Rex1 dependent production of ROS in mouse neutrophils stimulated with the chemoattractant fMLP. Purified neutrophils from wild type (*Prex^+/+^*, circles) or P-Rex deficient (*Prex^–/–^*, triangles) mice were treated with 1 µM PREX-in1 for 7 min (filled symbols), or were mock-treated (open symbols), prior to stimulation with 10 µM fMLP (*a*) or 500 nM PMA (*b*). Real-time ROS production was measured using a luminometer. Top panels show representative curves. The amount of ROS produced (area under the curve, AUC) in 2 min (a) or 10 min (b), respectively, and the height and timing of the peak of ROS production were quantified. Data are mean ± SEM of 5 experiments; each dot is the mean of one experiment. fMLP and PMA responses were tested using neutrophils from the same mice. Statistics are two-way ANOVA with Sidak’s multiple comparisons test.

Finally, to investigate the effects of PREX-in1 on a cell response dependent on endogenous P-Rex1, we measured ROS production in neutrophils from P-Rex deficient *Prex^−/−^* (*Prex1^−/−^Prex2^−/−^*) mice and control *Prex^+/+^* (*Prex1^+/+^Prex2^+/+^*) mice. As only P-Rex1 (not P-Rex2) is expressed in mouse neutrophils, this constitutes in effect a P-Rex1 deficient context. P-Rex1 was previously shown to be required for ROS production in neutrophils stimulated with the chemoattractant fMLP, but dispensable for receptor-independent ROS production induced with the PKC activator PMA [9–11]. Stimulation of mouse neutrophils with 10 μM fMLP induced rapid ROS production, which was lower in *Prex^−/−^* cells as expected. Treatment of *Prex^+/+^* neutrophils with 1 µM PREX-in1 for 7 min prior to the assay inhibited ROS production overall by 50%, both reducing the height of the ROS response and delaying it (Figure 8a). Crucially, PREX-in1 did not affect the ROS response in *Prex^−/−^* neutrophils, which shows that the inhibitor acts *via* P-Rex (Figure 8a). The PMA-induced ROS response, which was normal in *Prex^−/−^* neutrophils, as expected, was unaffected by PREX-in1 except for a small delay in the peak of the response in *Prex^+/+^* cells. Therefore, PREX-in1 inhibits GPCR-dependent ROS production in mouse neutrophils that depends on endogenous P-Rex, without affecting the integrity of the NADPH oxidase or P-Rex independent ROS production.

## Discussion

In this study, we developed PREX-in1 and derivative small molecules which inhibit P-Rex family Rac-GEFs with low micromolar efficacy through the catalytic DH domain. We show that PREX-in1 is selective for P-Rex1 and P-Rex2 over a panel of other Rho-GEFs and that it inhibits the P-Rex1-dependent spreading of endothelial cells and production of ROS in neutrophils. Hence, PREX-in1 and its derivatives will be useful research tools for the study of P-Rex function.

We used modelling to identify small-molecule compounds that might target the catalytic DH domain of P-Rex family Rac-GEFs. We searched for compounds that might mimic W56 peptide, as this peptide inhibited P-Rex1 to some extent, although at unfeasibly high concentrations, whereas neither NSC22766 nor EHop-016 did. This led to the development of PREX-in1 and several derivatives, the best of which are compounds #61 and #116. Use of these inhibitors with the isolated DH or DHPH domains of P-Rex1 and P-Rex2, as well as the full-length proteins, showed that they do indeed target the catalytic DH domains of P-Rex family GEFs, as they were designed to do. Compared to other Rho-GEF inhibitors, e.g. inhibitors for Tiam1, Trio-N, and DOCK2 [46,50,53], PREX-in1 and its best derivatives have lower IC_50_s (between 2 and 4.5 µM) and therefore better efficacy. The IC_50_ of PREX-in1 is 4.5 µM, as determined *in vitro*, although it was effective in cells at 1 µM. It is possible that a proportion of the recombinant P-Rex1 protein used *in vitro* may have been inactive but still able to bind PREX-in1, and that the actual IC_50_ of PREX-in1 is therefore lower, more similar to its effective concentration *in vivo*. In addition to good efficacy, PREX-in1 may also have greater specificity than other inhibitors. For example, NSC23766 inhibits both Tiam and Trio family Rac-GEFs [46], and EHop-016 targets both Tiam and Vav family Rac-GEFs [48], whereas PREX-in1 inhibits P-Rex1 and P-Rex2, but not Tiam1 or any of the Cdc42- or Rho-specific GEFs tested, nor indeed Rac. Structural work will be required in the future to confirm the exact site of PREX-in1 binding to the P-Rex DH domain and the mechanism of this specificity.

PREX-in1 inhibited the PDGF-stimulated spreading of PAE cells which expressed P-Rex1 but not that of control cells. Similarly, PREX-in1 inhibited the fMLP-stimulated production of ROS in wild type mouse neutrophils, but not P-Rex deficient neutrophils. Furthermore, PREX-in1 did not affect PMA-stimulated ROS production, which is P-Rex independent. Hence, PREX-in1 has on-target effects *in vivo*. PREX-in1 and its derivatives will therefore be useful for evaluating P-Rex function in cells that are short-lived, such as neutrophils, or terminally differentiated, such as neurons, or other cells types that cannot easily be manipulated by transfection, complementing genetic approaches.

We did not observe cytotoxic effects of PREX-in1 in HEK-293 cells, PAE cells or mouse neutrophils when used at 1 µM. However, PREX-in1 caused death of HEK-293 cells when used at 10 µM for 24 h. HEK-293 cells do express P-Rex1, but at quite low levels [60], suggesting that this effect of PREX-in1 may be off-target cytotoxicity. NSC23766, which caused cell death to a similar extent, is widely used and known to cause apoptosis in some but not all cell lines [61]. It remains unclear how much of that is due to the inhibition of Rac, as NSC23766 can affect cell responses even in the absence of Rac, for example in Rac1-deficient platelets [62]. Overall, it seems likely that long-term treatment with PREX-in1 induces a similar level of cytotoxicity as NSC23766. Cytotoxic effects of PREX-in1 and its derivatives remain to be investigated further before these compounds can be used to inhibit P-Rex Rac-GEFs in animal models of disease.

Recently, potential P-Rex1 inhibitors were reported by Cash *et al*. who searched for small molecules that can compete for PIP_3_ binding to the PH domain, aimed at inhibiting the PIP_3_-dependent activation of P-Rex1 [63]. Cash *et al*. used differential scanning fluorimetry, a technique which measures changes in the temperature at which a protein denatures as an indication of small-molecule binding. Three compounds were identified that had this effect on the isolated PH domain of P-Rex1. However, effects of these compounds on the Rac-GEF activity of Rex1 were not investigated, so it is unknown if they inhibit P-Rex1, or indeed any other GEFs. Moreover, unlike the headgroup of PIP_3_, these compounds also affected the denaturation temperature of PIP_3_ binding-deficient PH domain mutants, and they failed to co-crystalize with the PH domain. Hence, binding to the P-Rex1 PH domain remains to be corroborated. The compounds (at 30 µM) inhibited fMLP-stimulated neutrophil spreading [63]. This was possibly an off-target effect, as neutrophil spreading is mediated by Vav GEFs rather than P-Rex1 [9]. The compounds reduced the fMLP-stimulated activation of Rac2 in PLB-985 cells, although GEF-dependence was not evaluated. However, these data may need to be re-evaluated as similar Rac2 inhibition was shown for EGF-stimulated MCF7 breast cancer cells [63], which do not express Rac2 [64]. Finally, one of the compounds inhibited neutrophil migration to tail fin wounds in zebrafish larvae (at 100 µM), a response known to involve Rac2 [65,66], although again GEF-dependence was not investigated. Therefore, it remains unknown if the compounds identified by Cash *et al*. inhibit P-Rex. If further investigation confirms this, it will be useful to have both sets of inhibitors available, ours which block the DH domain, and therefore P-Rex activation by any upstream signal, and theirs which were designed to target PIP_3_-stimulated P-Rex activity.

The importance of P-Rex-Rac GEFs in pathophysiology, particularly cancer, together with the fact that these proteins are dispensable for development [5], make them attractive drug targets. Our rational and relatively small-scale, iterative approach to developing P-Rex inhibitors brought benefits. In particular, it allowed us to use the liposome-based GEF activity assay for testing compounds. The great advantage of this assay are the liposomes, which mimic the cell membrane and allow the use of fully post-translationally modified proteins, including prenylated GTPases, full-length GEFs and membrane-bound activators such as PIP_3_. However, the liposome-based assay is not scalable for high-throughput screening, as it is time-consuming and requires the use of radioactive ^35^S-GTPγS. Yet, PREX-in1 also worked in the mant-GTP GEF activity assay, and that platform may be useful for large-scale development of more sophisticated drug-like P-Rex inhibitors based on PREX-in1 in the future.

Drugs that target P-Rex Rac-GEFs would be particularly useful in the treatment of melanoma and breast cancer. P-Rex1 is overexpressed [16], and P-Rex2 is frequently mutated in human melanoma [27], and they are required for melanoma growth or metastasis in mice [16,27,28]. P-Rex1 is also overexpressed in luminal breast cancer, correlated with oestrogen receptor and ErbB2 expression, and as an effector of ErbB may be particularly important in ErbB2-positive breast cancer [25]. Depletion of P-Rex1 reduced mammary tumour growth in an orthotopic mouse model [25], and overexpression of P-Rex1 in mouse mammary tissue caused tumour initiation and metastasis [67]. P-Rex2 inhibits the tumour suppressor PTEN, stimulating the growth of breast cancer cells, and in cooperation with active PI3K, promotes growth factor-independent proliferation and transformation [42]. Moreover, PTEN inversely inhibits P-Rex2, and several tumour-associated P-Rex2 mutants escape this negative regulation in breast cancer cells [68]. Existing melanoma therapeutics can successfully shrink melanoma metastases, but only temporarily. Similarly, numerous therapies for breast cancer exist, reflecting the molecular heterogeneity of this cancer. More therapies for both diseases are in development. Drugs that target P-Rex family Rac-GEFs would add a new type of weapon to this armoury.

## Supplementary Material

Supplemental Material
