# Supplementary material for: Small-molecule inhibitors of P-Rex guanine-nucleotide exchange factors
Source: Small GTPases. 2022 Nov 7;13(1):307–26. doi: 10.1080/21541248.2022.2131313 (PMC9645260; doi:10.1080/21541248.2022.2131313)
Supplement: Supplemental Material [file KSGT_A_2131313_SM5360.zip › supplement/Lawson PREX1 inhibitor Supplemental Figure 1 Legend.docx]

**Supplemental Material**

**Small-Molecule Inhibitors of P-Rex Guanine-Nucleotide Exchange Factors**

Lawson CD^1^, Hornigold K^1^, Pan D^1^, Niewczas I^2^, Andrews S^3^, Clark J^2^, and Welch HCE^1,5^

^1^ Signalling Programme, ^2^ Biological Chemistry Facility, and  ^3^ Bioinformatics Facility, The Babraham Institute, Babraham Research Campus, Cambridge CB22 3AT, United Kingdom

**Legend to Supplemental Figure 1**

**Supplemental Figure Legend**

**Supplemental Figure 1. Pilot experiments which suggest that P-Rex1 Rac-GEF activity is inhibited by W56 peptide but not by NSC23766.** *(A)* Full-length recombinant EE-P-Rex1 (50 nM final concentration) was incubated with 200 µM NSC23766 for 30 min, or was mock-treated, before its Rac-GEF activity was measured by liposome-based GEF assay with prenylated EE-Rac2 as substrate (100 nM final) in the presence of liposomes that did or did not contain 10 µM PIP_3_. Rac2 activity is expressed as % of maximal GTP loading in the EDTA positive control. Data are mean ± range of duplicates from one pilot experiment representative of two. *(B)* P-Rex1 Rac-GEF activity was measured as in *(A)* after incubation of EE-P-Rex1 with the indicated concentrations of W56 peptide, except that 37 nM prenylated EE-Rac1 was used as substrate. The inhibition of P-Rex1 by W56 peptide is plotted using Rac-GEF activity without W56 peptide as 0% and Rac1 GTP-loading without P-Rex1 as 100%. Data are mean ± range of duplicates from one pilot experiment.
